# Prehospital pulse pressure and mortality of septic shock patients cared for by a mobile intensive care unit

**DOI:** 10.1186/s12873-023-00864-0

**Published:** 2023-08-25

**Authors:** Romain Jouffroy, Basile Gilbert, Jean Pierre Tourtier, Emmanuel Bloch-Laine, Patrick Ecollan, Josiane Boularan, Vincent Bounes, Benoit Vivien, Papa Gueye

**Affiliations:** 1 Intensive Care Unit, Ambroise Paré Hospital, Assistance Publique Hôpitaux Paris and Paris Saclay University, 9 avenue Charles De Gaulle, Boulogne-Billancourt, 92100 France; 2 Intensive Care Unit, Anaesthesiology, SAMU, Necker Enfants Malades Hospital, Assistance Publique - Hôpitaux Paris, Paris, France; 3 Centre de recherche en Epidémiologie et Santé des Populations - U1018 INSERM, Paris Saclay University, Villejuif, France; 4 Institut de Recherche bioMédicale et d’Epidémiologie du Sport - EA7329, INSEP - Paris University, Paris, France; 5 EA 7525 Université des Antilles, Fort de France, France; 6 Department of Emergency Medicine, SAMU 31, University Hospital of Toulouse, Toulouse, France; 7 Paris Fire Brigade, Paris, France; 8 Emergency Department, Cochin Hospital, Paris, France; 9 Emergency Department, SMUR, Hôtel Dieu Hospital - Assistance Publique - Hôpitaux Paris, Paris, France; 10 Intensive Care Unit, SMUR, Pitie Salpêtriere Hospital, 47 Boulevard de l’Hôpital, Paris - Assistance Publique - Hôpitaux Paris, Paris, 75013 France; 11 SAMU 31, Centre Hospitalier Intercommunal Castres-Mazamet, Castres, France; 12 SAMU 972, Centre Hospitalier Universitaire de Martinique, Fort-de-France Martinique, France; 13 EA 7525 University of the Antilles, Martinique, France

**Keywords:** Septic shock, Pulse pressure, Cardiac output, Prehospital setting, Association

## Abstract

**Background:**

Septic shock medical treatment relies on a bundle of care including antibiotic therapy and hemodynamic optimisation. Hemodynamic optimisation consists of fluid expansion and norepinephrine administration aiming to optimise cardiac output to reach a mean arterial pressure of 65mmHg. In the prehospital setting, direct cardiac output assessment is difficult because of the lack of invasive and non-invasive devices. This study aims to assess the relationship between 30-day mortality and (i) initial pulse pressure (iPP) as (ii) pulse pressure variation (dPP) during the prehospital stage among patients cared for SS by a prehospital mobile intensive care unit (MICU).

**Methods:**

From May 09th, 2016 to December 02nd, 2021, septic shock patients requiring MICU intervention were retrospectively analysed. iPP was calculated as the difference between systolic blood pressure (SBP) and diastolic blood pressure (DBP) at the first contact between the patient and the MICU team prior to any treatment and, dPP as the difference between the final PP (the difference between SBP and DBP at the end of the prehospital stage) and iPP divided by prehospital duration. To consider cofounders, the propensity score method was used to assess the relationship between (i) iPP < 40mmHg, (ii) positive dPP and 30-day mortality.

**Results:**

Among the 530 patients analysed, pulmonary, digestive, and urinary infections were suspected among 43%, 25% and 17% patients, respectively. The 30-day overall mortality rate reached 31%. Cox regression analysis showed an association between 30-day mortality and (i) iPP < 40mmHg; aHR of 1.61 [1.03–2.51], and (ii) a positive dPP; aHR of 0.56 [0.36–0.88].

**Conclusion:**

The current study reports an association between 30-day mortality rate and iPP < 40mmHg and a positive dPP among septic shock patients cared for by a prehospital MICU. A negative dPP could be helpful to identify septic shock with higher risk of poor outcome despite prehospital hemodynamic optimization.

## Introduction

Every year, more than 30 million people worldwide suffer from sepsis [1–3]. Sepsis is responsible for approximately 11 million deaths each year accounting for 20% of annual deaths [3] and almost 40% of all in-hospital deaths [4]. In 2016, the “sepsis 3” conference, the World Health Organization and the Centre for Disease Control and Prevention recommend early recognition, severity assessment and treatment instauration to decrease mortality of sepsis [5]. Indeed, during the last 40 years, sepsis overall mortality rate remains stable around 30% ranging from 15% for sepsis and 50% for septic shock, the most severe sepsis form [6–8].

From a pathophysiological point of view, an absolute and relative hypovolemia reflects the vascular sepsis consequences. Sepsis is characterized by the vascular tone decrease, traduced by micro, e.g., skin mottling, and macro-circulation alterations, e.g., hypotension. In order to correct both absolute and relative hypovolemia, to restore the vascular tone, and to ensure tissues perfusion [9, 10], the guidelines recommend an objective of a mean arterial pressure of at least 65 mmHg [11, 12] by fluid volume expansion within the first 3 h, and norepinephrine infusion in case of fluid expansion failure [5, 13, 14] aiming to optimise cardiac output and to ensure adequate tissues perfusion. However, undue fluid volume expansion results in a risk of fluid overload [15], independently associated with a poorer outcome, for example with septic shock mortality increase [16–20]. Cardiac output assessment can be performed by non-invasive approach, i.e., echocardiography, or invasive approach, i.e., Swan-Ganz catheterisation, both approaches, to date, are non-available in the prehospital daily practice. Pulse pressure (PP), e.g., the difference between systolic blood pressure (SBP) and diastolic blood pressure (DBP), is an indirect method of assessing cardiac output and an alternative for a non-invasive approach of cardiac output. Marik et al. previously reported that a PP less than 40mmHg indicates an impaired cardiac output [21].

To date most cases of sepsis (70%) occur outside hospital environment [22] with a median time to hospital admission around 60 min, to respect the treatment delay of sepsis guidelines for septic shock, the prehospital stage of care offers an opportunity to respect the delays while starting care early by prehospital caregivers [12, 23] [24]. Moreover, prompt prehospital and in-hospital hypotension correction improves septic shock survival [25–28]. Beyond sepsis origin source and antibiotic therapy, cardiac output and tissue perfusion optimization are daily questions in intensive care units in order to improve sepsis and septic shock outcome [11, 12]. In this way, PP is a parameter immediately available, non-invasive, reproductible and accessible since the prehospital setting where the resources are scarce. We hypothesized that PP, as a non-invasive surrogate of cardiac output, could be helpful for MICU physician daily practice, to early optimize cardiac output and tissue perfusion among septic shock patients.

Because, parameters variation is more informative than an isolated measure to assess the disease severity and the treatment effect, by similarity with the blood lactate clearance, for sepsis severity [29, 30] and treatment effect assessments [5, 30–33], we explored the relationship between pulse pressure variation (dPP, i.e., final prehospital PP – initial prehospital PP) and septic shock outcome, hypothesizing that, as shock index changes and lactate clearance during the prehospital stage, dPP may be an indirect tool for treatment effect assessment [5, 34, 35].

This study aims to assess the relationship between 30-day mortality and (i) initial pulse pressure (iPP) as (ii) dPP during the prehospital stage of care among patients cared for septic shock by a French mobile intensive care unit (MICU).

## Methods

### Patients

From May 09th, 2016 to December 02nd, 2021, patients with septic shock diagnosis presumed on clinical history, clinical signs and lactate measurement of available accordingly to the 2012 sepsis-2 conference [36] cared for by a prehospital MICU teams of one of 7 French hospital centres (Necker-Enfants malades Hospital, Lariboisière Hospital, La Pitié-Salpêtrière Hospital, Hôtel Dieu Hospital, APHP, Paris – France; The Paris Fire Brigade Paris, – France; The Toulouse University Health Centre, Toulouse – France and the Castres Hospital, Castres – France) were retrospectively included and patients care records were retrospectively analyzed in 2022. Patients younger than 18 years, and/or are pregnant, and/or with serious comorbid condition(s) with a not to be reanimated status known since pre-hospital setting were not included. Treatments management and strategy used to achieve a mean arterial pressure at the end of prehospital care were left to the MICU physician’s discretion.

Patients’ demographic characteristics, suspected prehospital origin of sepsis, initial prehospital (e.g., the first MICU contact), and final prehospital (e.g., at the end of prehospital stage) vital sign values (systolic blood pressure (SBP), diastolic blood pressure (DBP) and mean arterial pressure) were measured with French certified© non-invasive automated device in all centres (tool brands varied between centres), heart rate (HR), pulse oximetry (SpO2), respiratory rate (RR), body core temperature and Glasgow coma scale (GCS)), duration of prehospital care, and prehospital treatments (antibiotic therapy, fluid volume expansion, as well as catecholamine type and dose) were collected from MICU prehospital medical reports.

Hypertension, chronic cardiac failure (CCF), coronary heart disease (CHD), chronic renal failure (CRF), chronic obstructive pulmonary disease (COPD), diabetes mellitus, and history of cancer) [37] and immunosuppression defined by the existence of chronic alcoholism and/or human immunodeficiency virus infection were identified on MICU and in-hospital medical reports.

The length of stay (LOS) in the intensive care unit, in-hospital LOS, and the 30-day mortality status (alive or deceased) were retrieved from medical reports in case of in-hospital death or by patient and/or relatives phone call in case of hospital discharge. The Sequential Organ Failure Assessment (SOFA) score [38] was calculated 24 h after ICU admission.

### Ethical considerations

The Society of Anaesthesia and Intensive Care ethics committee on December 12th, 2017 (Ref number: IRB 00010254-2017-026) approved the study considering that the patient consent was waived for the participation in this retrospective study.

### Statistical analysis

Results are expressed as mean ± standard deviation for quantitative parameters with a Gaussian-distribution, as median with interquartile range [Q1-Q3] for parameters with a non-normal distribution and value with percentage for qualitative parameters. The main outcome was the 30-day mortality. Univariate and multivariate analyses were performed to evaluate the relationship between each covariate and 30-day mortality. Initial pulse pressure (iPP) was calculated by the difference between SBP and DBP at the first contact between the patients and the MICU team prior to any treatment. According to Marik et al. review [21], a threshold of 40mmHg was chosen to define a lowered cardiac output. Delta PP (dPP) was calculated by the difference between the final PP, the difference between SBP and DBP at the end of the prehospital stage, and iPP divided by prehospital duration (minutes). To consider cofounders, the propensity score (PS) method was used to assess the relationship between (i) iPP < 40mmHg, (ii) positive dPP and 30-day mortality. To reduce the effect of confounders on (i) iPP < 40mmHg, (ii) positive dPP and 30-day mortality, a propensity score matching was used to balance the differences in baseline characteristics between patients with (i) iPP < 40mmHg or (ii) positive dPP and those with (i) iPP ≥ 40mmHg or (ii) negative dPP. For iPP < 40mmHg, the propensity score, i.e., the probability of (i) iPP < 40mmHg was estimated using logistic regression based on potential confounders: age, sex, cancer history, CHD, CRF, diabetes mellitus, SOFA, hypertension, CCF, BMI, COPD and immunosuppression. For positive dPP, the propensity score, i.e., the probability of dPP > 0, was estimated using logistic regression based on potential confounders: antibiotic therapy administration, fluid expansion and norepinephrine administration during the prehospital setting, age, sex, cancer history, CHD, CRF, diabetes mellitus, SOFA, hypertension, CCF, BMI, COPD, and immunosuppression. Nearest neighbour matching method was used to match patients based on the logit of the propensity score [39]. The balance of covariates after matching was assessed by absolute mean differences with a considered acceptable threshold of 10% [40]. A survival analysis using Cox proportional hazards regression was used to compare 30-day mortality of patients with and without (i) iPP < 40mmHg, (ii) positive dPP in the propensity score–matched cohort. Proportional hazards assumption was verified for each Cox model variable by Kaplan Meier curve and log-rank test. Results are expressed by adjusted Hazard ratio (aHR) with 95% confidence interval [95 CI]. All tests were 2-sided.

R 3.4.2 software (http://www.R-project.org; the R Foundation for Statistical Computing, Vienna, Austria) was used for statistical analyses. A *p-value* < 0 0.05 defined statistical significance.

## Results

### Patient characteristics

Five hundred and thirty patients with septic shock cared for by a prehospital MICU team of one of 7 French hospital centres were analysed. Among them, 341 patients (65%) were male, and the mean age was 69 ± 15 years old (Table 1).

**Table 1:** Population characteristics. Results were expressed as mean and standard deviation or as median and interquartile range for quantitative parameters depending on distribution, and as an absolute value and percentage for qualitative parameters. The p-value corresponds to the comparison between deceased and living patients

	*Overall population (n = 530)*	*Living (n = 366)*	*Deceased (n = 164)*	*p value*
*Demographics*				
Age (years)	69 ± 15	68 ± 15	73 ± 14	**< 10** ^**− 3**^
Male gender	341 (64%)	243 (66%)	98 (60%)	0.141
BMI (kg.m^− 2^)	27.8 ± 37.5	29.3 ± 44.9	24.3 ± 6.2	**0.038**
Hypertension	230 (43%)	159 (43%)	71 (43%)	0.974
Chronic cardiac failure	134 (25%)	74 (20%)	60 (37%)	**< 10** ^**− 3**^
Coronary heart failure	104 (20%)	64 (17%)	40 (24%)	0.065
Diabetes Mellitus	151 (28%)	109 (30%)	42 (26%)	0.326
Cancer history	186 (35%)	116 (32%)	70 (43%)	**0.015**
COPD	79 (15%)	49 (13%)	30 (18%)	0.144
Chronic Renal Failure	75 (14%)	45 (12%)	30 (18%)	0.069
Immunosuppression	189 (36%)	120 (33%)	69 (42%)	**0.040**
***Prehospital***				
Initial SBP (mmHg)	97 ± 30	99 ± 30	93 ± 30	0.056
Initial DBP (mmHg)	58 ± 19	59 ± 19	55 ± 20	0.069
iPP (mmHg)	39 ± 17	40 ± 17	38 ± 18	0.200
iPP < 40mmHg	292 (55%)	198 (54%)	94 (57%)	0.491
Initial MAP (mmHg)	71 ± 22	72 ± 22	68 ± 22	0.064
Initial HR (beats.min^− 1^)	114 ± 28	115 ± 28	113 ± 31	0.463
Initial RR (movements.min^− 1^)	30 [22–36]	28 [22–35]	31 [25–38]	**0.007**
Initial pulse oximetry (%)	92 [85–96]	93 [87–96]	90 [83–95]	**0.006**
Initial body core temperature (°C)	38.3 [36.5–39.1]	38.4 [36.8–39.3]	38.1 [36.0–39.0]	**0.018**
Initial Glasgow coma scale	15 [12–15]	15 [13–15]	14 [11–15]	**0.002**
Initial blood lactate (mmol.l^− 1^)	5.8 ± 3.4	5.7 ± 3.3	6.3 ± 3.6	0.071
Fluid expansion (ml)	750 [500–100]	750 [500–1000]	750 [500–1000]	0.523
Norepinephrine administration	155 (29%)	104 (28%)	51 (31%)	0.530
Prehospital AB administration	132 (25%)	97 (27%)	35 (21%)	0.205
Prehospital duration (min)	71 ± 34	69 ± 33	74 ± 35	0.111
Final SBP (mmHg)	106 ± 25	109 ± 25	100 ± 24	**< 10** ^**− 3**^
Final DBP (mmHg)	62 ± 18	63 ± 18	60 ± 10	0.058
dPP (mmHg.min^− 1^)	0.07 ± 0.39	0.08 ± 0.39	0.02 ± 0.41	0.100
Final MAP (mmHg)	77 ± 19	78 ± 19	74 ± 19	**0.040**
Final HR (beats.min^− 1^)	107 ± 25	107 ± 25	109 ± 25	0.396
Final RR (movements.min^− 1^)	25 [19–30]	24 [18–30]	26 [20–34]	**0.011**
Final pulse oximetry (%)	97 [94–99]	97 [95–99]	97 [93–98]	**< 10** ^**− 3**^
Final body core temperature (°C)	38.1 [36.2–39.3]	38.0 [36.9–39.1]	38.9 [35.9–39.6]	**0.041**
Final Glasgow coma scale	15 [14–15]	15 [14–15]	14 [12–15]	**< 10** ^**− 3**^
Final blood lactate (mmol.l^− 1^)	4.2 ± 3.3	3.5 ± 2.8	5.7 ± 3.8	**< 10** ^**− 3**^
***Hospital***				
SOFA score	6 [3–9]	5 [3–8]	7 [4–10]	**< 10** ^**− 3**^
In-ICU length of stay (days)	4 [2–8]	4 [2–9]	3 [1–7]	**0.007**
In-hospital length of stay (days)	10 [5–18]	13 [8–21]	5 [2–11]	**< 10** ^**− 3**^

***Legend:*** *SBP = systolic blood pressure, DBP = diastolic blood pressure, iPP = initial pulse pressure, dPP = delta pulse pressure, MAP = mean arterial pressure, HR = heart rate, RR = respiratory rate, ICU = intensive care unit, SOFA = sequential organ failure assessment, COPD = chronic obstructive pulmonary disease, AB = antibiotic therapy, min = minutes.*

*Values in bold indicate a p-value < 0.05 between living and deceased patients*

Pulmonary, digestive and urinary infections were suspected in the prehospital setting for 43%, 25% and 17% patients, respectively (Table 2).

**Table 2:** Presumed septic shock origins. Data are expressed in absolute value and the corresponding percentages are indicated into brackets. Due to percentage rounding, the sum overpasses 100%

*Origin*	*n (percentage)*
Pulmonary	230 (43%)
Digestive	130 (25%)
Urinary	88 (17%)
Cutaneous	33 (6%)
Meningeal	11 (2%)
Gynaecological	3 (1%)
Ears nose throat	2 (0.5%)
Cardiovascular	2 (0.5%)
Unknown	31 (6%)

No significant difference in the prehospital stage duration, prehospital fluid expansion and antibiotic therapy was observed between patients who survived and those who died (Table 1).

Among the 132 patients (259%) who received antibiotic therapy prior to hospital admission, 74%were given 3rd generation cephalosporin among which 39% was with cefotaxime and 60% with ceftriaxone.

The median intensive care unit length of stay was 4 [2–8] days and the median length of stay in a hospital was 10 [5–18] days (Table 1).

The 30-day overall mortality reached 31%.

### Bivariate analysis

#### Initial pulse pressure (iPP)

A significant association between 30-day mortality and the following variables: cancer, prehospital initial SBP, SDP, mean arterial pressure, RR, norepinephrine, antibiotic therapy administration, prehospital final mean arterial pressure and RR for patients with a PPi < 40mmHg (Table 3).

**Table 3:** **Characteristics of patients with PPi ≥ 40mmHg and patients with PPi < 40mmHg**. Results were expressed as mean and standard deviation or as median and interquartile range for quantitative parameters depending on distribution, and as an absolute value and percentage for qualitative parameters. The p-value corresponds to the comparison between patients with PPi < 40mmHg and patients with PPi ≥ 40mmHg

	*PPi < 40mmHg (n = 292)*	*PPi* ≥ *40mmHg (n = 238)*	*p value*
*Demographics*			
Age (years)	69 ± 14	70 ± 15	0.750
Male gender	190 (65%)	151 (63%)	0.699
BMI (kg.m^− 2^)	29.7 ± 5.2	25.4 ± 6.4	0.239
Hypertension	125 (43%)	133 (56%)	0.763
Chronic cardiac failure	75 (26%)	179 (75%)	0.814
Coronary heart failure	64 (22%)	198 (83%)	0.141
Diabetes Mellitus	77 (26%)	164 (69%)	0.232
Cancer history	116 (40%)	168 (71%)	**0.013**
COPD	45 (15%)	204 (86%)	0.718
Chronic Renal Failure	38 (13%)	201 (84%)	0.406
*Immunosuppression*	110 (38%)	159 (67%)	0.285
***Prehospital***			
Initial SBP (mmHg)	98 ± 18	117 ± 28	**< 10** ^**− 3**^
Initial DBP (mmHg)	52 ± 17	64 ± 20	**< 10** ^**− 3**^
Initial MAP (mmHg)	62 ± 17	82 ± 23	**< 10** ^**− 3**^
Initial HR (beats.min^− 1^)	113 ± 28	116 ± 29	0.371
Initial RR (movements.min^− 1^)	28 [20–35]	31 [24–38]	**0.012**
Initial pulse oximetry (%)	92 [85–96]	92 [85–96]	0.152
Initial body core temperature (°C)	38.3 [36.4–39.0]	38.4 [36.9–39.2]	0.134
Initial Glasgow coma scale	15 [13–15]	15 [12–15]	0.127
Initial blood lactate (mmol.l^− 1^)	6.2 ± 3.6	5.6 ± 3.2	0.103
Fluid expansion (ml)	1000 [500–1200]	750 [500–1000]	0.154
Norepinephrine administration	106 (36%)	189 (79%)	**< 10** ^**− 3**^
Prehospital AB administration	84 (29%)	190 (80%)	**0.023**
Prehospital duration (min)	67 ± 34	74 ± 34	0.017
Final SBP (mmHg)	102 ± 23	111 ± 26	**< 10** ^**− 3**^
Final DBP (mmHg)	61 ± 18	64 ± 18	0.111
Final MAP (mmHg)	75 ± 19	79 ± 20	**0.019**
Final HR (beats.min^− 1^)	107 ± 24	108 ± 26	0.379
Final RR (movements.min^− 1^)	24 [19–30]	25 [20–32]	**0.089**
Final pulse oximetry (%)	97 [95–99]	97 [94–99]	0.219
Final body core temperature (°C)	38.2 [36.1–39.4]	38.1 [36.3–39.1]	0.932
Final Glasgow coma scale	15 [14–15]	15 [13–15]	0.204
Final blood lactate (mmol.l^− 1^)	4.2 ± 3.2	4.3 ± 3.5	0.576
***Hospital***			
SOFA score	6 [4–9]	5 [2–8]	0.104
In-ICU length of stay (days)	4 [2–8]	4 [1–8]	0.940
In-hospital length of stay (days)	10 [5–17]	10 [5–18]	0.923

*Legend: SBP = systolic blood pressure, DBP = diastolic blood pressure, MAP = mean arterial pressure, HR = heart rate, RR = respiratory rate, ICU = intensive care unit, SOFA = sequential organ failure assessment, COPD = chronic obstructive pulmonary disease, AB = antibiotic therapy, min = minutes*

*Values in bold indicate a p-value < 0.05 between patients with PPi < 40mmHg and patients with PPi ≥ 40mmHg*

#### Delta pulse pressure (dPP)

A significant association between 30-day mortality and the following variables: prehospital initial SBP, SDP, mean arterial pressure, RR, SpO2, norepinephrine, antibiotic therapy administration, prehospital duration, prehospital final SBP and mean arterial pressure and GCS for patients with a positive dPP (Table 4).

**Table 4:** **Characteristics of patients with positive delta pulse pressure (dPP > 0) and patients with negative delta pulse pressure (dPP < 0)**. Results were expressed as mean and standard deviation or as median and interquartile range for quantitative parameters depending on distribution, and as an absolute value and percentage for qualitative parameters. The p-value corresponds to the comparison between patients with positive delta pulse pressure (dPP > 0) and patients with negative delta pulse pressure (dPP < 0)

	*dPP < 0 (n = 208)*	*dPP > 0 (n = 321)*	*p value*
*Demographics*			
Age (years)	70 ± 15	69 ± 15	0.445
Male gender	138 (66%)	203 (63%)	0.466
BMI (kg.m^− 2^)	24.8 ± 5.5	29.7 ± 4.8	0.219
Hypertension	86 (41%)	143 (45%)	0.468
Chronic cardiac failure	52 (25%)	82 (26%)	0.888
Coronary heart failure	41 (20%)	63 (20%)	0.981
Diabetes Mellitus	57 (27%)	94 (29%)	0.640
Cancer history	79 (38%)	107 (33%)	0.274
COPD	30 (14%)	49 (15%)	0.791
Chronic Renal Failure	37 (18%)	38 (12%)	0.057
Immunosuppression	70 (34%)	119 (37%)	0.423
***Prehospital***			
Initial SBP (mmHg)	110 ± 31	89 ± 26	**< 10** ^**− 3**^
Initial DBP (mmHg)	61 ± 20	56 ± 19	**0.006**
Initial MAP (mmHg)	77 ± 23	67 ± 21	**< 10** ^**− 3**^
Initial HR (beats.min^− 1^)	115 ± 29	114 ± 28	0.601
Initial RR (movements.min^− 1^)	30 [24–39]	28 [22–35]	**0.005**
Initial pulse oximetry (%)	91 [84–96]	92 [85–96]	**0.011**
Initial body core temperature (°C)	38.3 [36.4–39.1]	38.4 [36.9–39.1]	0.378
Initial Glasgow coma scale	15 [12–15]	15 [13–15]	0.698
Initial blood lactate (mmol.l^− 1^)	5.8 ± 3.3	5.8 ± 3.4	0.919
Fluid expansion (ml)	700 [500–1000]	925 [500–1238]	0.368
Norepinephrine administration	46 (22%)	108 (34%)	**0.005**
Prehospital AB administration	40 (19%)	92 (29%)	**0.015**
Prehospital duration (min)	64 ± 31	75 ± 35	**< 10** ^**− 3**^
Final SBP (mmHg)	98 ± 23	111 ± 25	**< 10** ^**− 3**^
Final DBP (mmHg)	63 ± 19	62 ± 17	0.437
Final MAP (mmHg)	74 ± 20	78 ± 19	**0.025**
Final HR (beats.min^− 1^)	110 ± 26	106 ± 24	0.067
Final RR (movements.min^− 1^)	25 [20–32]	24 [19–30]	0.051
Final pulse oximetry (%)	97 [94–98]	97 [95–99]	0.060
Final body core temperature (°C)	38.2 [36.0–39.2]	38.1 [36.8–39.0]	0.702
Final Glasgow coma scale	15 [13–15]	14 [14–15]	**0.041**
Final blood lactate (mmol.l^− 1^)	4.4 ± 3.4	4.1 ± 3.2	0.459
***Hospital***			
SOFA score	6 [3–9]	5 [3–9]	0.902
In-ICU length of stay (days)	3 [1–7]	4 [2–8]	0.121
In-hospital length of stay (days)	10 [4–18]	11 [6–18]	0.053

*Legend: SBP = systolic blood pressure, DBP = diastolic blood pressure, MAP = mean arterial pressure, HR = heart rate, RR = respiratory rate, ICU = intensive care unit, SOFA = sequential organ failure assessment, COPD = chronic obstructive pulmonary disease, AB = antibiotic therapy, min = minutes*

*Values in bold indicate a p-value < 0.05 between positive delta pulse pressure (dPP > 0) and patients with negative delta pulse pressure (dPP < 0).*

### Survival analysis

#### Initial pulse pressure < 40mmHg

**Fig. 1:**
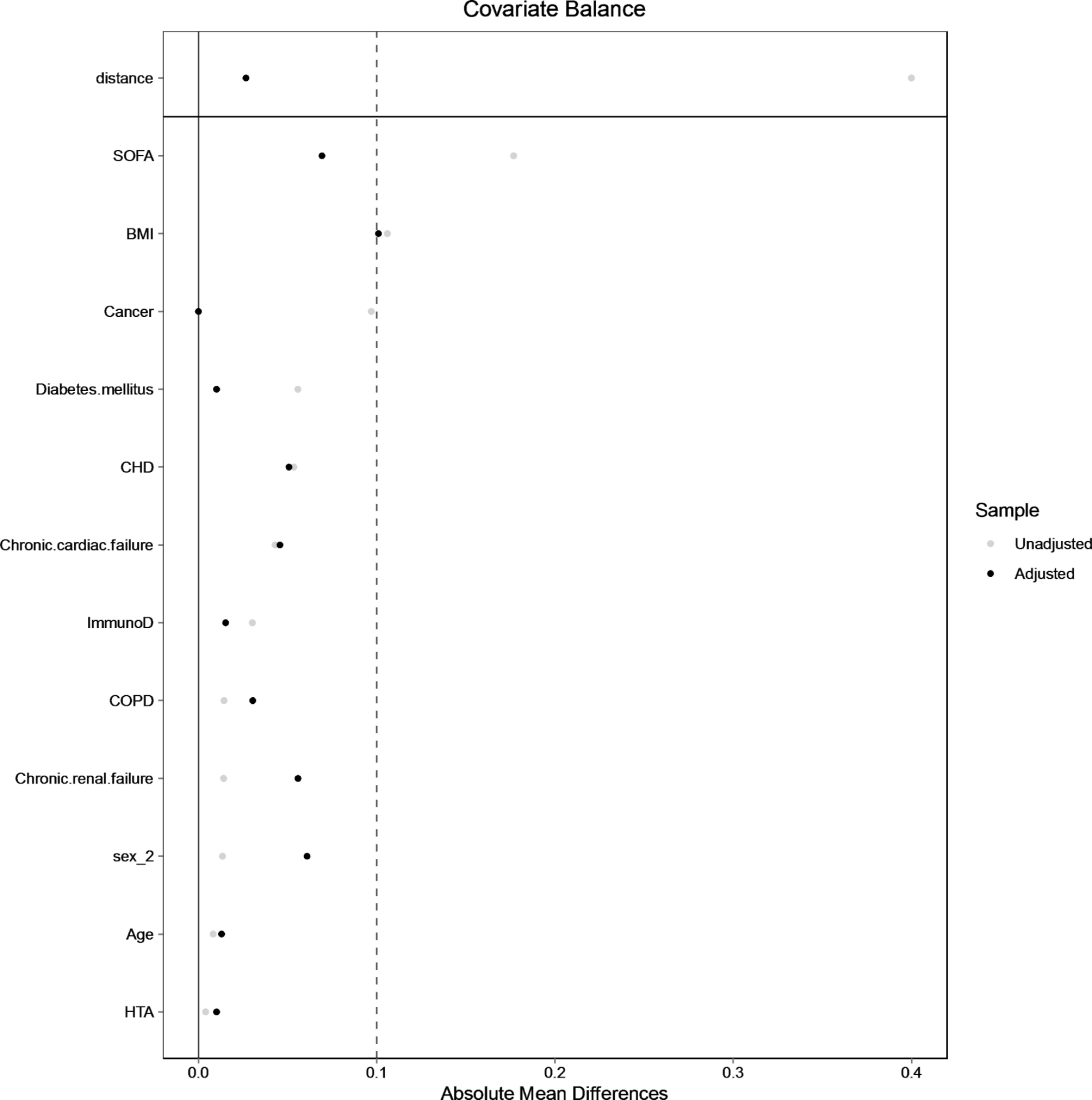
Absolute mean differences between patients with iPP < 40mmHg and those with iPP ≥ 40mmHg after matching

The matched population consists of 88 controls, i.e., iPP ≥ 40mmHg and 197 cases, i.e., iPP < 40mmHg. The absolute mean differences between cases and controls after propensity score matching is depicted in Fig. 1.

#### Positive delta pulse pressure

**Fig. 2:**
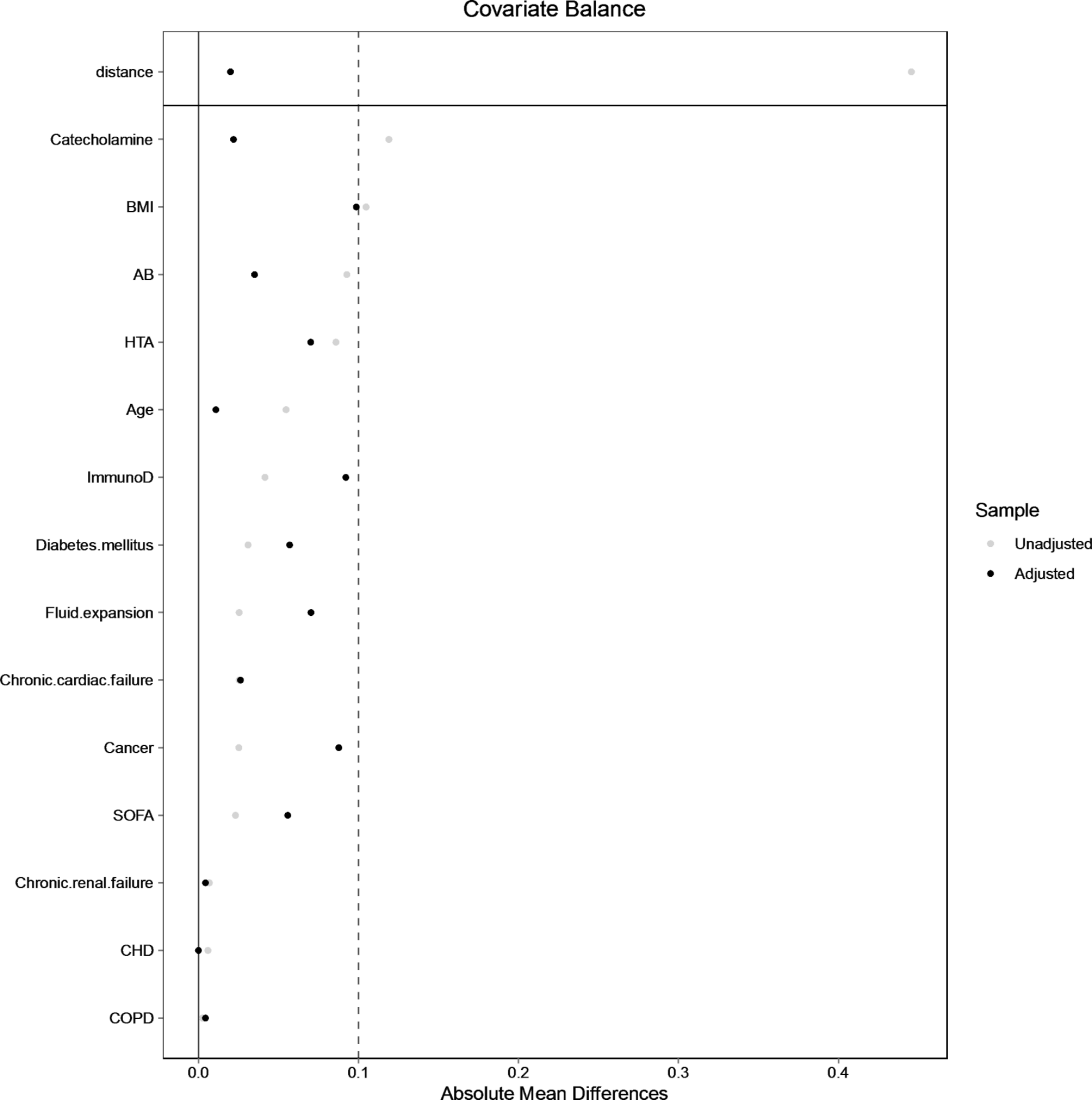
Absolute mean differences between patients with positive dPP and those without negative dPP after matching

The matched population consists of 77 controls, i.e., negative delta pulse pressure and 228 cases, i.e., positive pulse pressure. The absolute mean differences between cases and controls after propensity score matching is depicted in Fig. 2.

**Fig. 3:**
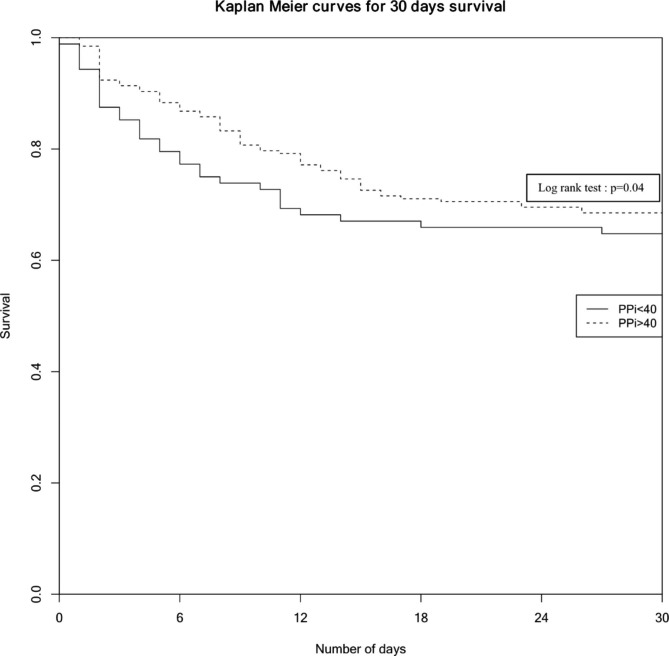
**Kaplan Meier curves for 30-days survival between patients with iPP < 40mmHg and those with iPP ≥ 40mmHg after matching**

Cox regression analysis after matching showed an association between 30-day mortality and iPP < 40mmHg: aHR = 1.61 [1.03–2.51], log rank test p = 0.04. Kaplan Meier curves depict differences on 30-day survival in both subgroups after adjustment of confounders (Fig. 3).

**Fig. 4:**
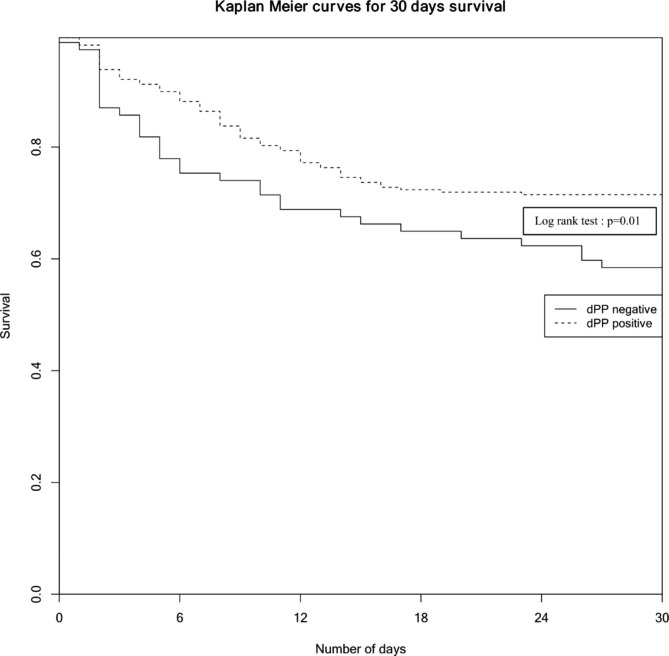
**Kaplan Meier curves for 30-days survival between patients with positive dPP and those without negative dPP after matching**

Cox regression analysis after matching showed an association between 30-day mortality and a positive dPP: aHR = 0.56 [0.36–0.88], log rang test p = 0.01. Kaplan Meier curves depict differences on 30-day survival in both subgroups after adjustment of confounders (Fig. 4).

## Discussion

An iPP < 40mmHg and a negative dPP are associated with 30-day mortality increase in patients suffering from septic shock cared for by a prehospital MICU. A negative prehospital dPP could be helpful for MICU physicians’ daily practice to identify septic shock patients with higher risk of poor outcome despite prehospital hemodynamic optimization.

The associated sepsis systemic response inflammatory syndrome results in both absolute and relative hypovolemia. Macro circulatory alterations, e.g. low blood pressure and/or cardiac output decrease, and microcirculatory alterations, e.g. hyperlactatemia or skin mottling, parameters [41] are associated with sepsis poorer outcome [42, 43]. To restore the tissues and organs’ perfusion, by restoring a sufficient cardiac output and mean arterial pressure, the international sepsis guidelines recommend early fluid expansion and norepinephrine infusion, when mean arterial pressure remains lower than 65 mmHg [5, 12, 23]. Because the negative association between the fluid resuscitation volumes, in other words the net fluid balance [15–20], and sepsis mortality is established, the optimal treatment aims to find the right equilibrium between fluid volume requirement and fluid volume overload [16–20, 44]. Since the prehospital and in hospital norepinephrine infusion in combination with, but not without [45], fluid resuscitation is feasible without increasing adverse effects [46]; in 2019, the Surviving Sepsis Campaign advocates the use of vasopressors even during the fluid resuscitation to reach and maintain a mean arterial pressure ≥ 65mmHg within the first hour after sepsis recognition [12]. The beneficial effects of norepinephrine are partly mediated by the cardiac output increase, mediated by the norepinephrine beta-2 agonist effect, and/or by the vascular tone increase mediated by the norepinephrine alpha-1 agonist effect [47].

Previous studies reported an association between septic shock outcome and clinical signs, biomarkers and severity scores [38, 42, 43, 48–50]. However, in the prehospital setting, only clinical signs, few biomarkers [51] and qSOFA, whose validity remains under debate [52–58], are currently available. For severity assessment, to date, lactatemia remains the best biomarker [59, 60], available in the prehospital setting [61], also allowing a dynamic approach based on lactatemia clearance for treatment effect assessment [5, 34]. To bypass biomarkers’ and qSOFA limits, capillary refill time, skin mottling score and shock index usefulness were described for septic shock severity assessment [35, 62–64]. iPP and dPP are in line with other clinical signs reflecting the severity of septic shock and the treatment effect of prehospital care. To the best of our knowledge, this study is the first to describe the relationship between iPP, dPP and 30-day mortality of septic shock patients cared for by a prehospital MICU.

### Study limitations

Our study suffers from several limitations. From a methodological point of view, the bias from misclassification of covariates cannot be excluded as data were collected from prehospital and in-hospital medical reports. Moreover, because data abstraction was collected by a single investigator, the data accuracy can be compromised [65]. The statistical analysis performed does not allow any causal conclusion between iPP < 40mmHg, positive PP and 30-day mortality. In this study, we only included adults, consequently our conclusions are not directly transposable to a pediatric population. This is a retrospective study; because no therapeutic goal was a priori defined, we cannot define which mean arterial pressure was targeted nor when was prescribed norepinephrine administration before or after fluid expansion failure. We cannot exclude that the specificity of the French prehospital emergency medical service could affect the results’ external validity.

However, this study results suggest that iPP reflects septic shock severity and in a similar manner to lactate clearance or shock index variation. dPP could be used for treatment effect assessment and could be helpful to MICU physicians’, in their daily practice, to early optimize septic shock patients’ cardiac output and tissue perfusion.

## Conclusion

An iPP < 40mmHg and a positive dPP are associated with 30-day mortality in patients with septic shock cared for by prehospital MICU. Despite prehospital hemodynamic optimization, a negative prehospital dPP may identify patients with higher risk of poorer outcome. Further studies are needed to evaluate if prehospital iPP < 40mmHg and positive dPP alone or combined with clinical scores and/or biomarkers could affect the prehospital triage decision-making process.

## Data Availability

The dataset analyzed during the current study are not publicly available because their containing information that could compromise the privacy of *research* participants but are available from the corresponding author on reasonable request.

## Abbreviations

MICU: Mobile intensive care unit
aHR: Adjusted hazard ratio
SAMU: Urgent Medical Aid Service
SMUR: Mobile Emergency and Resuscitation Service
SBP: Systolic blood pressure
DBP: Diastolic blood pressure
HR: Heart rate
SpO2: Pulse oximetry
RR: Respiratory rate
GCS: Glasgow coma scale
LOS: Length of stay
SOFA: Sequential Organ Failure Assessment
qSOFA: Quick Sequential Organ Failure Assessment
PP: Pulse pressure
CCF: Chronic cardiac failure
CHD: Coronary heart disease
CRF: Chronic renal failure
COPD: Chronic obstructive pulmonary disease
BMI: Body mass index
